# *INF2*‐Related Charcot–Marie–Tooth Disease in a Japanese Cohort: Genetic and Clinical Insights

**DOI:** 10.1002/acn3.70205

**Published:** 2025-09-23

**Authors:** Chikashi Yano, Masahiro Ando, Yujiro Higuchi, Jun‐Hui Yuan, Akiko Yoshimura, Takahiro Hobara, Risa Nagatomo, Fumikazu Kojima, Yu Hiramatsu, Satoshi Nozuma, Tomonori Nakamura, Yusuke Sakiyama, Chika Matsuoka, Toru Yamashita, Takashi Kimura, Ayako Miyazaki, Chinatsu Kinjo, Kenji Yokochi, Nanami Yamanaka, Nozomu Matsuda, Tomoki Suichi, Yoshiyuki Hanaoka, Haruka Kojima, Kenichi Todo, Hiroyuki Ishiura, Jun Mitsui, Shoji Tsuji, Hiroshi Takashima

**Affiliations:** ^1^ Department of Neurology and Geriatrics Kagoshima University Graduate School of Medical and Dental Sciences Kagoshima Japan; ^2^ Department of Neurology Okayama University Graduate School of Medicine, Dentistry and Pharmaceutical Sciences Okayama Japan; ^3^ Department of Neurology Hyogo Medical University Hyogo Japan; ^4^ Department of Clinical Genetics Hyogo Medical University Hyogo Japan; ^5^ Department of Pediatrics Toyohashi Municipal Hospital Aichi Japan; ^6^ Department of Neurology and Clinical Neuroscience Yamaguchi University Graduate School of Medicine Yamaguchi Japan; ^7^ Department of Neurology Fukushima Medical University School of Medicine Fukushima Japan; ^8^ Department of Neurology Graduate School of Medicine, Chiba University Chiba Japan; ^9^ Department of Pediatrics Kurashiki Central Hospital Okayama Japan; ^10^ Department of Neurology Tokyo Women's Medical University Tokyo Japan; ^11^ Department of Precision Medicine Neurology Graduate School of Medicine, The University of Tokyo Tokyo Japan; ^12^ Department of Neurology The University of Tokyo Hospital Tokyo Japan; ^13^ Institute of Medical Genomics International University of Health and Welfare Chiba Japan

**Keywords:** Charcot‐Marie‐Tooth disease, focal segmental glomerulosclerosis, INF2, inherited peripheral neuropathies, neuropathy

## Abstract

**Background:**

*INF2* mutations cause focal segmental glomerulosclerosis (FSGS) and Charcot–Marie–Tooth disease (CMT). Accurate genetic diagnosis is critical, as *INF2*‐related FSGS is typically resistant to immunotherapy yet rarely recurs after transplantation, and its associated neuropathy can mimic treatable immune‐mediated disorders such as chronic inflammatory demyelinating polyradiculoneuropathy (CIDP).

**Methods:**

We performed a multicenter study investigating 3329 Japanese patients with inherited peripheral neuropathies/CMT who underwent gene panel sequencing or whole‐exome analysis between 2007 and 2024. Clinical data, including electrophysiological assessments, were obtained from the patients' medical records.

**Results:**

We identified six pathogenic *INF2* variants in eight patients, all of which were located within the diaphanous inhibitory domain. Structural modeling revealed clustering of variants near the diaphanous autoregulatory domain‐binding pocket, which is critical for INF2 autoinhibition. Clinically, all cases were sporadic, with a median age at neurological onset of 9 years. All patients exhibited lower limb weakness, and 6/8 (75%) had sensory disturbances. All patients also developed kidney dysfunction, with 7/8 (88%) progressing to end‐stage renal disease at a median age of 15 years. Furthermore, all patients showed demyelinating neuropathy, and 2/8 (25%) received immunotherapy due to suspected immune‐mediated neuropathy.

**Conclusion:**

Although *INF2* variants are a rare cause of CMT in Japan, they should be considered in pediatric patients with demyelinating neuropathy and early‐onset proteinuria, even in the absence of a family history. Blood and urine tests assessing renal dysfunction can provide guidance for appropriate genetic testing.

## 1 Introduction

Charcot–Marie–Tooth disease (CMT) is the most common type of inherited peripheral neuropathy (IPN). This clinically and genetically heterogeneous condition is characterized by progressive motor and sensory deficits, foot deformities, and reduced tendon reflexes. Some patients also present with extraneural features, such as deafness or retinopathy. In 2011, it was discovered that *INF2* variants caused focal segmental glomerulosclerosis (FSGS) and CMT [1].

FSGS is a major cause of steroid‐resistant nephrotic syndrome, in which impaired glomerular filtration leads to proteinuria and progressive renal dysfunction, often culminating in end‐stage renal disease (ESRD). A Japanese study reported that 34% of patients with FSGS carried pathogenic gene variants, of which approximately 20% involved *INF2* [2]. *INF2* encodes a formin protein that plays a role in actin polymerization and cytoskeletal regulation. Functional disruption of INF2 affects organelle dynamics in podocytes and Schwann cells, contributing to FSGS and CMT pathology [3]. Although immunosuppressive therapy and kidney transplantation are standard FSGS treatments, inherited forms, such as *INF2*‐related FSGS, often respond poorly to immunotherapy, whereas they are less likely to recur after transplantation [4]. Furthermore, *INF2*‐related neuropathy mimics immune‐mediated disorders, such as chronic inflammatory demyelinating polyradiculoneuropathy (CIDP), potentially leading to inappropriate immunotherapy [5]. Therefore, early genetic diagnosis is crucial for optimizing renal and neurological therapeutic strategies.

*INF2*‐related CMT is relatively rare in Japan, and its clinical and electrophysiological features are insufficiently characterized. Thus, this study aimed to investigate the clinical, electrophysiological, and genetic characteristics of Japanese patients with IPNs who carry pathogenic *INF2* variants, identified through a large‐scale genetic screening cohort.

## 2 Materials and Methods

### 2.1 Enrollment Criteria

We performed detailed clinical and genetic analyses on Japanese patients participating in a nationwide genetic study on IPNs/CMT. All patient records and assessments were obtained from the medical facilities involved and used to review clinical and electrophysiological data. Fluorescence in situ hybridization or multiple ligation‐dependent probe amplification was used to rule out *PMP22* (*peripheral myelin protein 22*) duplication/deletion in patients with demyelinating‐type neuropathies.

### 2.2 Genetic Analysis

Between 2007 and 2014, DNA samples from patients clinically diagnosed with IPN/CMT were screened using in‐house gene panel sequencing targeting IPN/CMT‐related genes. Sequencing was performed using DNA microarrays (2007–2012; Affymetrix, Santa Clara, CA, USA) or the Illumina MiSeq platform (2012–2014; Illumina, San Diego, CA, USA). Notably, *INF2* was not included in any of these panels. However, 485 samples without a definitive genetic diagnosis underwent subsequent whole‐exome sequencing that included *INF2* analysis. From 2014 to September 2024, the updated IPN/CMT‐related gene panels included *INF2*, and sequencing was performed on an Ion Proton System (Thermo Fisher Scientific, Waltham, MA, USA).

The sequence data were aligned to the NCBI37/hg19 human reference genome. Variant calling was performed using Burrows–Wheeler Aligner and SAMtools software, and variants were annotated using CLC Genomics Workbench software (Qiagen, Hilden, Germany) and an in‐house script. All potentially pathogenic variants were confirmed by Sanger sequencing. Previously reported variants, including those in *INF2*, were verified by consulting the Human Gene Mutation Database Professional database. These methods have been previously described [6].

### 2.3 Three‐Dimensional (3D) Structure Modeling of the *INF2* Diaphanous Inhibitory Domain (DID)

To investigate the structural context of the identified *INF2* variants, we used the AlphaFold2_mmseqs2 notebook (default parameters) via the Google Colaboratory platform to predict a 3D model of the human INF2 DID, which includes an autoinhibitory domain within diaphanous formins [7, 8].

## 3 Results

### 3.1 Genetic Findings and Structural Modeling of *INF2*

**FIGURE 1:**
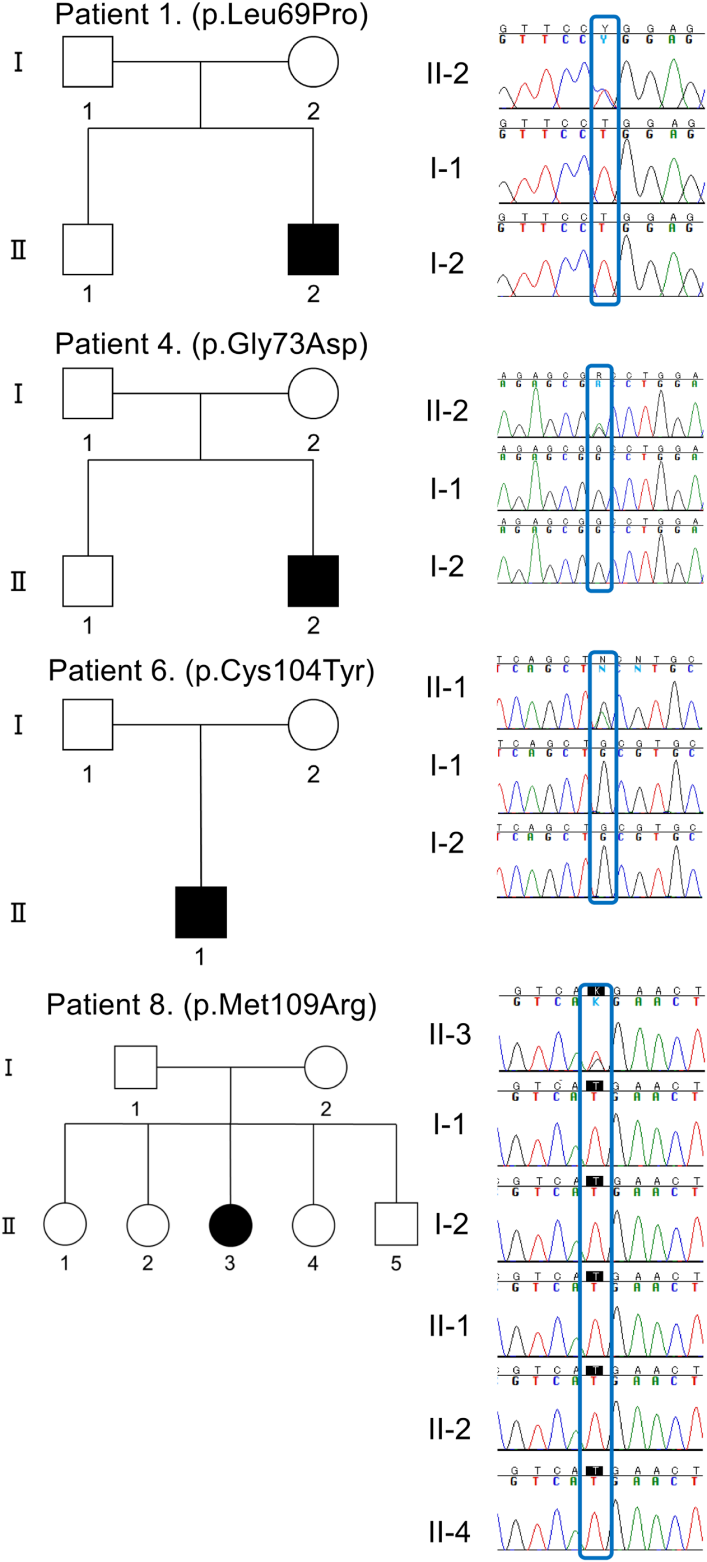
Four pedigrees of CMT families with *INF2* variants. Genotypes associated with the *INF2* variants are shown for each case. The genetic data for patient 2 has been previously reported (figure not shown, see reference [9]).

Among the study cohort (*N* = 3329), we identified 6 *INF2* (NM_022489.4) missense variants in 8 unrelated cases (whole‐exome sequencing, *n* = 4; targeted gene panel sequencing, *n* = 4). All variants have been previously described: c.206T>C, p.Leu69Pro (*n* = 2); c.218G>A, p.Gly73Asp (*n* = 2); c.230T>C, p.Leu77Pro (*n* = 1); c.311G>A, p.Cys104Tyr (*n* = 1); c.312C>G, p.Cys104Trp (*n* = 1); and c.326T>G, p.Met109Arg (*n* = 1). Segregation analyses were available for five pedigrees (patients 1, 2, 4, 6, and 8), and all variants in these cases (p.Leu69Pro, p.Gly73Asp, p.Cys104Tyr, and p.Met109Arg) were confirmed as de novo (Figure 1). The genetic findings of patient 2 have been previously reported [9].

**FIGURE 2:**
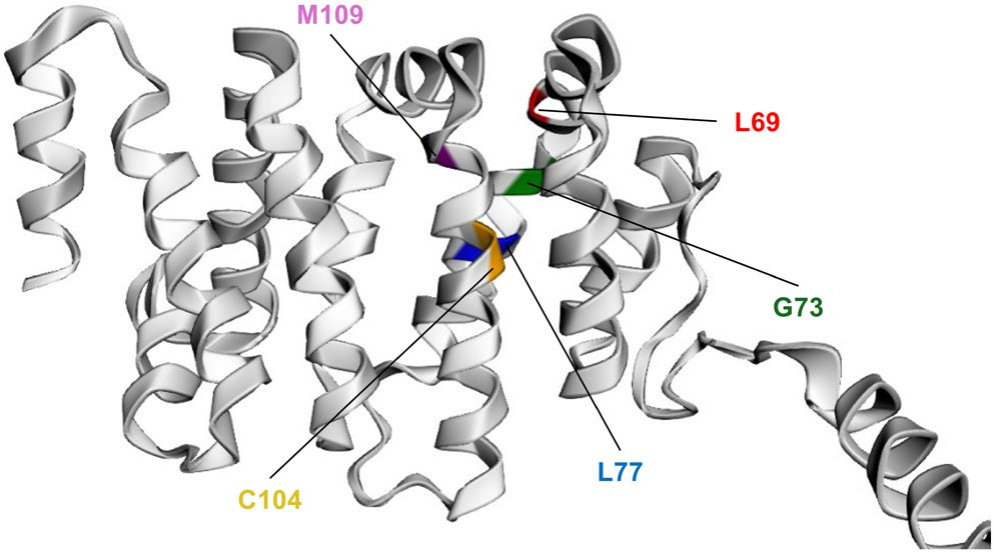
Structural localization of identified *INF2* variants within the DID. 3D model of the human INF2 DID generated using AlphaFold2 via the ColabFold: AlphaFold2_mmseqs2 notebook. Five distinct positions within the DID harboring the six identified *INF2* variants are shown, colored as follows: Leu69 (red), Gly73 (green), Leu77 (blue), Cys104 (orange), and Met109 (purple). The Cys104 position includes two variants: Cys104Trp and Cys104Tyr.

Exon 2 forms a critical part of the DID of the INF2 protein. Structural analysis revealed that all six *INF2* variants were located across five distinct positions within exon 2, resulting in five amino acid substitutions (Figure 2).

### 3.2 Clinical Findings

Table 1 summarizes the clinical characteristics of the eight patients with *INF2* variants, of which six (75%) were male and two (25%) female. None of the patients had a family history of neuropathy or renal dysfunction.

**TABLE 1:** Clinical characteristics of *INF2*‐related CMT.

Characteristic	Patient							
	1	2^a^	3^a^	4	5	6	7	8
Variant	c.206T>C p.Leu69Pro	c.206T>C p.Leu69Pro	c.218G>A p.Gly73Asp	c.218G>A p.Gly73Asp	c.230T>C p.Leu77Pro	c.311G>A p.Cys104Tyr	c.312 C>G p.Cys104Trp	c.326T>G p.Met109Arg
Sex	M	M	M	M	F	M	M	F
Age at exam (years)	15	35	21	20	23	8	50	30
Family history	Sporadic	Sporadic	Sporadic	Sporadic	Sporadic	Sporadic	Sporadic	Sporadic
Initial neurological symptom	Walking disability	Frequent falling	Poor motor skills	Frequent falling	Abnormal running	Falls, gait change	Foot deformity, drop foot	Frequent falling
AAN (years)	10	13	11	8	12	5	7	7
AAP (years)	10	10	9	8	NA	14	12	NA
ESRD age (years)	16	17	13	14	12	Not yet	15	15
Dialysis age (years)	−	17	13	14	−	−	15	15
Transplant age (years)	16	35	−	15	12	−	15	15
Hypertension	NA	+	+	+	NA	NA	+	−
Immunotherapy	−	+^b^	+^c^	−	−	−	+^d^	−
UL weakness	−	+	+	+	+	−	+	−
UL proximal MMT	5	5	4	5	5	5	5	5
UL distal MMT	5	2	2	4	4	5	2	5
LL weakness	+	+	+	+	+	+	+	+
LL proximal MMT	5	5	4	5	3	5	4	NA
LL distal MMT	2	1	2	1	1	2	1	2
Pes cavus	NA	+	+	+	+	+	+	+
Lower leg atrophy	+	+	+	+	+	+	+	+
Sensory disturbance	−	+	+	+	+	−	+	+
Superficial sensation	−	−	−	Decreased	Decreased	−	Decreased	NA
Deep sensation	−	Decreased	Decreased	Decreased	Decreased	−	Decreased	NA
DTR (PTR/ATR)	Normal	Absent	−/−	Decreased	−/−	−/−	−/−	+/−
Pathological reflex	−	−	−	−	NA	−	−	NA
Deafness	−	+	−	+	−	+	+	−
Autonomic symptoms	−	Constipation	−	−	NA	−	−	NA
Other findings	−	−	Nerve hypertrophy	Tremor	Tremor	−	Scoliosis, cataract	−

*Note:* −, Absent/no; +, Present/yes.

Abbreviations: AAN, age at neurological symptom; AAP, age at proteinuria; ATR, Achilles tendon reflex; DTR, deep tendon reflex; ESRD, end‐stage renal disease; F, female; LL, lower limb; M, male; MMT, manual muscle testing; NA, not available or not assessed; PTR, patellar tendon reflex; UL, upper limb.

^a^ Deceased patient.

^b^ Intravenous immunoglobulin treatment for neuropathy.

^c^ Prednisolone for suspected chronic inflammatory demyelinating polyradiculoneuropathy.

^d^ Prednisolone for focal segmental glomerulosclerosis.

The median age of onset of neurological symptoms was 9 years (interquartile range [IQR], 7–11.25 years; *n* = 8). All patients showed lower limb muscle weakness, and 5 (63%) also had upper limb muscle weakness. All patients had lower leg atrophy, and pes cavus was present in the 7 (88%) patients evaluated for this feature. Sensory disturbances were noted in 6/8 (75%) patients (deep and superficial sensory impairment: *n* = 3; deep sensory impairment, *n* = 2). Tendon reflexes were absent in 6 (75%) patients, decreased in 1 (12.5%), and normal in 1 (12.5%). None of the patients showed lateral asymmetry in motor weakness, sensory disturbance, or tendon reflexes. Hearing loss was observed in 4 (50%) patients. Patient 4 was the only case for whom the age of onset was clearly documented, with sensorineural hearing loss at 6 years old preceding the onset of proteinuria and neurological symptoms at 8 years old. None of the patients had retinopathy. Based on an initial suspicion of immune‐mediated neuropathy, 2 (25%) patients were treated with immunotherapy, including prednisolone and intravenous immunoglobulin. Three patients underwent nerve biopsies, revealing reduced myelinated fiber density with evidence of segmental demyelination and secondary axonal degeneration in all cases, and onion bulb formations in one case.

All eight patients exhibited renal involvement, with a median age of onset of 9.5 years (IQR, 9–12 years; *n* = 6) for proteinuria. One patient had no renal dysfunction at the time of genetic testing but later developed FSGS during follow‐up. Seven patients were diagnosed with FSGS, and one was diagnosed with infantile nephronophthisis. In the one case with a detailed biopsy report (Patient 4), 13 glomeruli were sampled; light microscopy showed mesangial proliferation in 6 glomeruli without crescents, PAS highlighted focal hyalinosis consistent with segmental sclerosis, type IV collagen α2/α5 staining showed a preserved pattern, and electron microscopy revealed no thinning or thickening of the glomerular basement membrane.

Seven of the eight patients progressed to ESRD and underwent renal replacement therapy, including dialysis (*n* = 5) or kidney transplantation (*n* = 6). The median age at ESRD onset was 15 years (IQR, 13–16 years; *n* = 7). One patient received steroid therapy for renal dysfunction, which was discontinued due to a lack of efficacy. Among the seven patients with available follow‐up data, two died suddenly in their 30s, but the direct cause was unclear.

### 3.3 Electrophysiological Findings

**FIGURE 3:**
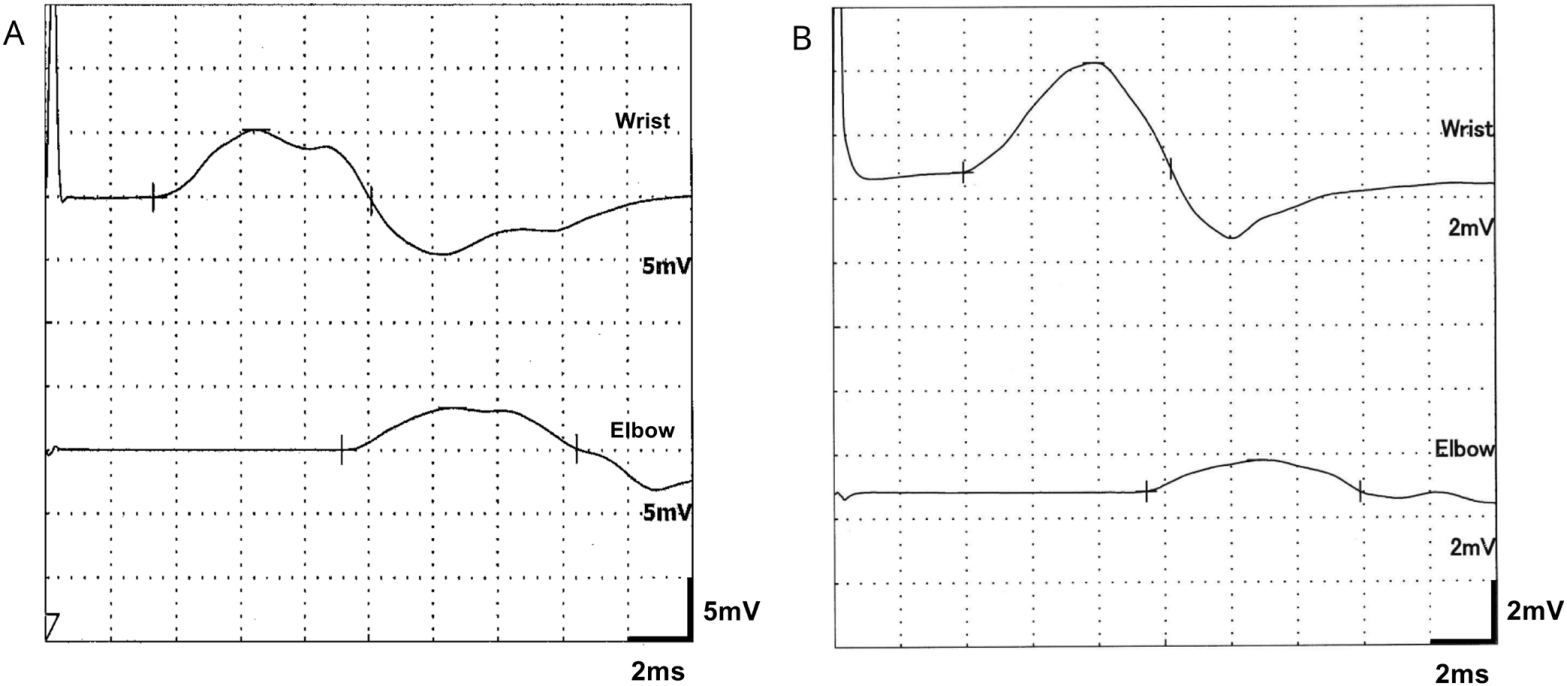
Nerve conduction study findings in two patients with conduction block. (A) Ulnar nerve in patient 4 (35% conduction block). (B) Median nerve in patient 6 (71% conduction block).

We assessed the electrophysiological findings in the eight patients with *INF2* variants. The median motor nerve conduction velocity in the upper limbs was 27.5 m/s (IQR, 23.9–31.6), and all patients were classified as having demyelinating‐type neuropathy (assessed using the median nerve in seven patients and the ulnar nerve in one patient, whose median nerve was undetectable) (Table 2). Reduced or absent compound muscle action potential (CMAP) amplitudes in the median nerve were present in 5/8 (63%) patients (median, 3.8 mV; IQR, 0.53–7.26). In the ulnar nerve, 3/5 (60%) patients showed reduced CMAP amplitudes (median, 4.0 mV; IQR, 0.33–5.68). All eight patients exhibited reduced or undetectable CMAP amplitudes in the tibial nerve (median, 0.16 mV; IQR, 0.01–0.35). Additionally, 3/8 (38%) patients had a conduction block of ≥ 30% in either the median or ulnar nerve (Figure 3). Regarding sensory nerves, all patients exhibited reduced or absent sensory nerve action potential (SNAP) amplitudes in the median, ulnar, and sural nerves. Notably, SNAPs from the sural nerves were undetectable in all patients.

**TABLE 2:** Motor and sensory nerve conduction studies in *INF2*‐related CMT.

Nerve conduction study results			Patient							
			1	2	3	4	5	6	7	8
Motor	Median	dCMAP (mV)	7.8	0.54	0.486	4.2	7.08	3.4	0	9.4
	Median	pCMAP (mV)	3.07	0.54	0.346	3.6	5.06	0.98		8.7
	Median	MCV (m/s)	26	23.9	18.3	33.3	32	31.5		23.8
	Ulnar	dCMAP (mV)	NE	NE	0.647	4	6.26	NE	0.02	5.1
	Ulnar	pCMAP (mV)			0.369	2.6	4.96		0.05	4.5
	Ulnar	MCV (m/s)			20.1	33.5	34		29	23.9
	Tibial	dCMAP (mV)	0.359	0	0.041	0.19	1.79	0.344	0	0.009
	Tibial	pCMAP (mV)	0.237		0.0227	0.09	0	0.156		0
	Tibial	MCV (m/s)	25.3		11.9	21.5		18.5		
Sensory	Median	SNAP (μV)	0	0	0	4	1	4.5	0	1.8
	Median	SCV (m/s)				29.5	26	39.9		26.7
	Ulnar	SNAP (μV)	NE	NE	0	3	3	NE	1	0
	Ulnar	SCV (m/s)				41.4	29		29.6	
	Sural	SNAP (μV)	0	0	0	0	0	0	0	0
	Sural	SCV (m/s)								

*Note:* Reference values: median nerve distal CMAP amplitude, > 3.1 mV; median nerve MCV, > 49.6 m/s; ulnar nerve distal CMAP amplitude, > 6.0 mV; ulnar nerve MCV, > 50.1 m/s; tibial nerve distal CMAP amplitude, > 4.4 mV; tibial nerve MCV, > 41.7 m/s; median nerve SNAP amplitude, > 5.0 μV; median nerve SCV, > 47.2 m/s; ulnar nerve SNAP amplitude, > 6.9 μV; ulnar nerve SCV, > 46.8 m/s; sural nerve SNAP amplitude, > 5.0 μV; sural nerve SCV, > 40.8 m/s.

Abbreviations: dCMAP, distal compound muscle action potential amplitude; MCV, motor nerve conduction velocity; NE, not examined; pCMAP, proximal compound muscle action potential amplitude; SCV, sensory nerve conduction velocity; SNAP, sensory nerve action potential amplitude.

Needle electromyography was performed in patient 7, revealing long‐duration, high‐amplitude motor unit potentials and reduced recruitment in both the upper and lower limb muscles. Active denervation signs, including fibrillation potentials and fasciculation potentials, were also observed.

## 4 Discussion

This study is the largest cohort‐based analysis of *INF2*‐related CMT in Japan, identifying IPNs/CMT in 8/3329 patients. We comprehensively characterized the clinical and genetic features of these patients with *INF2*‐related CMT. They exhibited early‐onset peripheral neuropathy during childhood and relatively early‐onset renal dysfunction, progressing to ESRD in most cases. Electrophysiologically, they demonstrated demyelinating patterns with reduced conduction velocities, with a subset showing conduction blocks.

It has been established that patients with CMT may also present with nephrotic syndrome. In 2011, it was reported that *INF2* variants were present in 75% of patients with both FSGS and CMT [1]. *INF2* encodes a member of the diaphanous‐related formin family, which regulates actin polymerization and depolymerization. Cytoskeletal disruption caused by INF2 dysfunction has been suggested to cause structural abnormalities of the endoplasmic reticulum and mitochondria, contributing to glomerular barrier breakdown in the kidney and myelination failure in Schwann cells [3]. In Schwann cells, INF2 colocalizes with and interacts with CDC42 and myelin‐and‐lymphocyte protein (MAL); disease‐associated variants cause INF2 mislocalization and disruption of the INF2–MAL–CDC42 pathway, thereby impairing actin dynamics and providing a mechanistic basis for primary Schwann cell demyelination [1, 3]. Since the initial reports, additional cases have been described, revealing that the *INF2* variant location determines whether FSGS alone or FSGS with CMT develops. Disease‐causing *INF2* variants predominantly cluster within exons 2–4, which encode the DID. Notably, variants causing both FSGS and CMT are concentrated in exon 2 (Leu57‐Glu184), whereas those causing isolated FSGS tend to cluster in exons 3–4 (Glu184‐Leu245) [2, 10]. All six variants identified in our study cohort were located in exon 2, and all patients presented with both FSGS and CMT. This observation is highly consistent with previous structural and mutational analyses mapping the pathogenic variants associated with FSGS and CMT to the central hydrophobic diaphanous autoregulatory domain (DAD)‐binding pocket within the INF2 DID. This specific region is essential for the autoinhibitory regulation of INF2 activity, mediated by its interaction with the DAD [2, 10]. The clustering of our identified variants within this functionally significant exon further highlights the importance of DID integrity for proper INF2 function.

Notably, all eight cases in our cohort were sporadic. Segregation analyses performed in five pedigrees confirmed de novo mutations in all cases, despite the autosomal dominant inheritance pattern of *INF2*‐related CMT. A previous study has suggested that de novo mutations are more common in patients with both CMT and FSGS than in those with FSGS alone [11]. Two possible mechanisms may explain this observation. First, individuals with combined CMT and FSGS often exhibit more severe motor dysfunction than those with FSGS alone and also progress to ESRD at a younger age. In our cohort, 7/8 patients developed ESRD during their teenage years, whereas in a Japanese cohort of patients with INF‐related FSGS without CMT—most of whom were in their 20s or older—only 1/7 progressed to ESRD. These two factors—severe motor and renal impairment—may contribute to reduced reproductive fitness and, consequently, a higher proportion of de novo cases [11, 12]. Second, all variants in our cohort were located in exon 2 of *INF2*, which lies within a CpG island (UCSC Genome Browser: https://genome.ucsc.edu/) and has a GC content of approximately 68% (Ensembl Genome Browser: https://asia.ensembl.org/). CpG islands and GC‐rich regions are associated with mutational hotspots, including sites of de novo variants [13]. Thus, these sequence‐specific properties of *INF2* exon 2 may contribute to the high frequency of de novo mutations observed in this region.

Clinically, all patients exhibited childhood onset with a slowly progressive, length‐dependent neuropathy characterized by distal‐predominant muscle atrophy and weakness, sensory disturbance, areflexia, and pes cavus, consistent with the typical phenotype of CMT. No central nervous system or autonomic manifestations were documented. Hearing loss occurred in 50% (4/8), which may serve as a potential diagnostic clue. Nevertheless, variable expressivity was evident even among carriers of the same variant, as reflected by discordant hearing loss and heterogeneity in the severity of distal weakness. Electrophysiologically, all patients with *INF2* variants (excluding those with unrecordable potentials) exhibited motor nerve conduction velocities ≤ 38 m/s, indicating a demyelinating pattern. Although *INF2*‐related CMT has been reported as a form of dominant intermediate CMT type E, none of the patients in our cohort showed intermediate conduction velocities, and all findings were consistent with a demyelinating type [1]. Moreover, all patients showed length‐dependent weakness, predominantly affecting the distal lower limbs, which is a hallmark of CMT. A distal CMAP amplitude < 1 mV in the tibial nerve was observed in 7/8 (87.5%) patients, and the remaining case also showed low amplitudes. Sural SNAPs were unrecordable in all cases. These findings support the presence of length‐dependent neuropathy on electrophysiological testing [14]. Distal short‐segment studies at the wrist/ankle were not performed; thus, a contribution from distal conduction block cannot be excluded, although the overall pattern remains most consistent with demyelination with superimposed length‐dependent axonal loss.

However, conduction block, typically a hallmark of acquired demyelinating neuropathies such as CIDP, was observed in 37.5% of cases, despite being considered atypical in CMT [15, 16]. These findings indeed fulfilled the electrodiagnostic criteria for CIDP, and two patients were initially suspected of having acquired neuropathies and were treated with immunotherapy [17]. Differentiating between CIDP and CMT can be challenging [5], the absence of a family history in all cases further contributed to the diagnostic challenge. Thus, urinalysis and renal function tests should be considered in pediatric patients suspected of CIDP, with the presence of proteinuria or renal dysfunction prompting genetic testing for *INF2* variants.

In our cohort, 7/8 (88%) patients progressed to ESRD in their teenage years and underwent renal transplantation or dialysis. Renal histopathology in *INF2*‐associated FSGS typically shows glomerular tuft collapse with focal segmental sclerosis and thinning of the glomerular basement membrane; clinically, these changes correlate with the development of proteinuria and eventual renal failure [18]. Although immunotherapy is occasionally used for treating FSGS, its efficacy in the hereditary form is limited, and progression to renal failure is common. One patient in our cohort received immunotherapy for FSGS but eventually required renal transplantation. Although FSGS recurrence after transplantation is a recognized concern, hereditary FSGS, including *INF2*‐related FSGS, is associated with a lower recurrence risk [19]. If *INF2*‐related FSGS is diagnosed early, renal protective strategies can be initiated at an earlier stage, potentially delaying progression to ESRD and improving long‐term renal outcomes. Importantly, anticipating ESRD enables timely planning for living donor kidney transplantation, facilitating more optimal donor selection and the implementation of long‐term treatment strategies. Thus, from the perspective of FSGS management, the early genetic diagnosis of *INF2* variants is clinically significant.

In our cohort, all patients with *INF2* variants developed renal dysfunction, and none presented with isolated CMT. By contrast, among eight patients with *INF2* variants identified in a Japanese proteinuria cohort, only one (Gly73Val) had CMT [12]. Among the six patients in our series with clearly documented timing of proteinuria onset, four developed proteinuria concurrently with neuropathic symptoms, whereas the remaining two—both carrying p.Trp104—developed proteinuria more than 5 years after neuropathy onset, consistent with a previous report [20]. These findings suggest that the clinical symptoms of *INF2*‐related disease, including the presence or absence of CMT, FSGS onset, and their timing, may be variant‐dependent. Thus, recognizing such genotype–phenotype correlations is crucial for accurate diagnosis, counseling, and long‐term care. Early genetic diagnosis should prompt longitudinal monitoring of proteinuria and renal function, which may facilitate timely intervention.

This study has several limitations. Clinical data were collected across multiple institutions, resulting in differences in data availability and completeness. Additionally, differences in electrophysiological procedures between institutions may have introduced technical variability, potentially affecting the interpretation and diagnostic accuracy. Distal conduction block was not systematically assessed, which may have led to underestimation of its frequency and limited the distinction between primary demyelination and secondary length‐dependent axonal loss.

In conclusion, although *INF2*‐related CMT is relatively rare in Japan, it should be considered in pediatric patients with demyelinating neuropathy and early‐onset renal dysfunction, particularly if CIDP is suspected. Urinalysis and renal function tests are essential for the diagnostic workup, and if persistent proteinuria or renal dysfunction is detected, genetic testing for *INF2* should be performed. A timely and accurate diagnosis will help avoid treatments that are unlikely to be effective, reduce unnecessary invasive procedures, and guide appropriate management strategies for both neuropathy and renal disease.

## Author Contributions

C. Yano, M. Ando, Y. Higuchi, and H. Takashima conceived and designed the study. C. Yano, M. Ando, Y. Higuchi, J.‐H. Yuan, T. Hobara, R. Nagatomo, F. Kojima, Y. Hiramatsu, S. Nozuma, T. Nakamura, Y. Sakiyama, C. Matsuoka, T. Yamashita, T. Kimura, A. Miyazaki, C. Kinjo, K. Yokochi, N. Yamanaka, N. Matsuda, T. Suichi, Y. Hanaoka, H. Kojima, K. Todo, and H. Ishiura contributed to the analysis and verification of the clinical data. C. Yano, M. Ando, J.‐H. Yuan, A. Yoshimura, J. Mitsui, and S. Tsuji contributed to the genetic data. J.‐H. Yuan performed proofreading. C. Yano drafted the original manuscript. All coauthors approved the final version.

## Ethics Statement

This study was approved by the Institutional Review Board of Kagoshima University. It was performed in accordance with the ethical standards laid down in the 1964 Declaration of Helsinki and its later amendments.

## Consent

Informed consent was obtained from all patients and their family members before study participation.

## Conflicts of Interest

The authors declare no conflicts of interest.

## Data Availability

The datasets used in our study are not readily available due to ethical and privacy restrictions. Requests should be directed to the corresponding author.
